# Case Report: Refractory Cytopenia With a Switch From a Transient Monosomy 7 to a Disease-Ameliorating del(20q) in a *NHEJ1*-Deficient Long-term Survivor

**DOI:** 10.3389/fimmu.2022.869047

**Published:** 2022-06-24

**Authors:** Fiona Poyer, Raúl Jimenez Heredia, Wolfgang Novak, Petra Zeitlhofer, Karin Nebral, Michael N. Dworzak, Oskar A. Haas, Kaan Boztug, Leo Kager

**Affiliations:** ^1^ St. Anna Children’s Hospital, Department of Pediatrics and Adolescent Medicine, Medical University of Vienna, Vienna, Austria; ^2^ St. Anna Children’s Cancer Research Institute (CCRI), Vienna, Austria; ^3^ Ludwig Boltzmann Institute for Rare and Undiagnosed Diseases, Vienna, Austria; ^4^ Center for Molecular Medicine Center for Molecular Medicine (CeMM) Research Center for Molecular Medicine of the Austrian Academy of Sciences, Vienna, Austria; ^5^ Labdia, Labordiagnostik, Vienna, Austria

**Keywords:** NHEJ1, refractory cytopenia, monosomy 7, del(20q), myelodysplastic syndrome, NHEJ1-deficiency

## Abstract

We report the case of a male Pakistani patient with a pathogenic homozygous loss of function variant in the non-homologous end-joining factor 1 (*NHEJ1*) gene. The growth retarded and microcephalic boy with clinodactyly of both hands and hyperpigmentation of the skin suffered from recurrent respiratory infections. He was five and a half years old when he came to our attention with refractory cytopenia and monosomy 7. Hematopoietic stem cell transplantation was considered but not feasible because there was no suitable donor available. Monosomy 7 was not detected anymore in subsequent bone marrow biopsies that were repeated in yearly intervals. Instead, seven and a half years later, a novel clone with a del(20q) appeared and steadily increased thereafter. In parallel, the patient’s blood count, which had remained stable for over 20 years without necessitating any specific therapeutic interventions, improved gradually and the erythropoiesis-associated dysplasia resolved.

## Introduction

Inherited bone marrow failure (IBMF) syndromes are genetically heterogeneous hematopoietic stem cell disorders that impede the adequate production of one or more blood cell lineages and consequently predispose affected individuals to the development of myelodysplastic syndromes (MDS) as well as myeloid malignancies (1–3). The responsible genetic defects often produce recognizable syndromes, whose main features are microcephaly, growth retardation as well as inconsistent other physical malformations and organ abnormalities. Such germline alterations comprise pathogenic variants in genes that encode for transcription factors such as GATA2, RUNX1 and ETV6, products that are involved in telomere maintenance (dyskeratosis congenita), ribosomal biogenesis (Blackfan-Diamond anemia) and maturation (Shwachman-Diamond syndrome; SDS), DNA maintenance and repair (Fanconi anemia), protein folding and trafficking (severe congenital neutropenia) as well as in the regulation of cell proliferation and apoptosis (SAMD9/9L) (3–9). In many instances characteristic clinical, laboratory and hematologic parameters alone will already suffice to identify the respective syndrome. Nevertheless, the large number of not only genes but also of possible types of pathogenic variants that need to be considered even in such well-defined syndromes requires a thorough molecular genetic clarification to obtain a precise diagnosis. In case of overlapping symptoms and/or if no pathogenic sequence abnormalities are found, an even broader screening approach with whole exome sequencing may be necessary to identify rarer or even previously not considered causes of such diseases.

Herein we report the extraordinary disease development in a patient with distinctive yet originally difficult to interpret phenotypic features, in whom, after a long and challenging diagnostic odyssey, we finally succeeded to secure a homozygous pathogenic variant in the *NHEJ1* gene as the responsible germ line defect.

## Case Report

The now 26-year-old patient [case #17; Table 2 in our previous publication (10)] is the son of consanguineous Pakistani parents, who were healthy and had normal blood counts. One sister was healthy, a second sister suffered from hepatitis C, but had normal blood counts. He had been prone to infections, primarily recurrent bronchitis, since birth and was first seen in our clinic when he was five and a half years old with fever and coughing. He was growth retarded and had a microcephaly (<3rd percentile), clinodactyly of both hands and hyperpigmented skin above the knees and elbows. Apart from his microcephaly, imaging of the head, the thorax and the abdomen remained inconspicuous. Screening for causative infectious agents, including *Mycobacterium tuberculosis*, was unrewarding. Immunological analyses that were performed during the course of the disease revealed IgA deficiency and B-lymphocytopenia (Details are provided in **Supplementary Table 1**). Hematologic analysis at first presentation revealed a white blood cell count of 1.47 x10^9^/L with an absolute neutrophil count of 0.76 x10^9^/L and an absolute lymphocyte count of 0.47 x10^9^/L, a hemoglobin level of 9.2 g/dL, MCV of 75fl, and a platelet count of 109 x10^9^/L. Data on the long-term course of hematological parameters are provided in **Supplementary Figure 1**. A bone marrow (BM) examination disclosed reduced cellularity of all three lineages with an erythropoiesis-restricted dysplasia that, together with the presence of a fluorescence *in situ* hybridization (FISH)-verified monosomy 7 in 18% of the analyzed nuclei, was consistent with the diagnosis of a hypocellular myelodysplastic syndrome in form of a refractory cytopenia (11, 12). Although the appearance of monosomy 7 in IBMF is strongly indicative of an underlying SAMD9/9L or GATA2 deficiency, these disease-promoting germline causes were not known at that time (6, 13, 14).

We discounted the most likely causes of the patient’s problems, namely Fanconi anemia, Nijmegen breakage syndrome and dyskeratosis congenita with an originally negative diepoxybutane (DEB) breakage analysis in the one, molecular testing in the other and based on clinical parameters in the latter, respectively. Another DEB test that was performed at the age of 14 years showed an elevated chromosome breakage (4.78, normal <0.6) but without the pathognomonic Fanconi anemia-specific chromatid exchange figures. Moreover, a cell cycle analysis of the patient’s skin fibroblasts, which was kindly performed in the Department of Human Genetics, University of Würzburg, Würzburg, Germany, lacked the otherwise typical G2 cell cycle blockage (15). Normal pancreatic laboratory parameters also excluded a less likely Shwachman-Diamond syndrome and lymphocyte immunophenotyping the presence of paroxysmal nocturnal hemoglobinuria clones that are seen in up to 38% of refractory cytopenia cases (16, 17). Since the appearance of monosomy 7 in patients with such syndromic features and pancytopenia often precedes and forecasts the transformation into myeloid malignancy, the current EWOG-MDS 2006 protocol (NCT00662090) recommends as the treatment of choice to perform a hematopoietic stem cell transplantation (HSCT), which was not feasible in this case because we did not find a suitable donor. However, contrary to all odds, his blood counts remained stable at this low level over the following years, and he did not even require any transfusions. He had only a mild obstructive ventilation disorder and continued to suffer from recurrent but well-manageable pulmonary infections. Although we did not find the clone with the monosomy 7 anymore in the follow-up BM examination three months later, another clone with a deletion of the long arm of chromosome 20, del(20q), emerged seven and a half years later, when the patient was 13 years old. The respective FISH analyses were performed with a del(20q)-specific dual-color probe set (leicabiosystems.com). Subsequent BM examinations that were executed in yearly intervals thereafter showed that this abnormal clone steadily increased from originally 15% to 90% within the following six years and then dropped again to 68% a year later, at which point the erythropoiesis-restricted dysplasia also had resolved. Nonetheless, even two years later, we found that 34% of the peripheral blood cells still descended from this del(20q) clone, even though the patient was in good clinical condition and with a notably improved white blood cell count of 3.06 x10^9^/L, an absolute neutrophil count of 1.27 x10^9^/L, an absolute lymphocyte count of 1.53 x10^9^/L, a hemoglobin level of 11.6 g/dL and a platelet count of 82 x10^9^/L. The hematological parameters, however, display a fluctuating pattern during course (**Supplementary Figure 1**).

## Genetic Analyses

**Figure 1:**
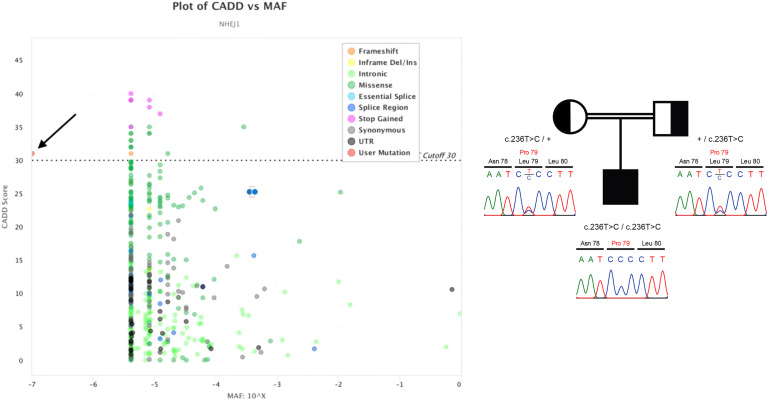
Left: CADD versus minor allele frequency (MAF) plot of all known *NEHJ1* sequence variants visualized by PopViz (18). The horizontal axis shows the MAF scores and the vertical axis the CADD ones. The specific types of the various sequence variants, which were collected from the gnomAD r2.0.2 database (https://gnomad.broadinstitute.org/gene/ENSG00000187736?dataset=gnomad_r2_1), are color-coded, and the pathogenic variant of our patient is indicated by the black arrow. Right: The patient’s homozygous *NEHJ1* gene pathogenic variant (NM_024782.2:c.236T>C, p.Leu79Pro) was inherited from his heterozygous parents.

When our patient was 21 years old, we finally succeeded to identify the genetic cause of his physical and hematological problems with our next-generation targeted sequencing hematology panel. This approach uncovered a unique and hitherto undescribed pathogenic homozygous missense variant (NM_024782.2:c.236T>C, p.Leu79Pro; GRCh37) in exon 3 of the *NHEJ1* (“non-homologous end-joining factor 1”, also known as *XLF* “*XRCC4*-like factor” or Cernunnos, OMIM *611290) gene on chromosome 2(q35) (10). The pathogenic variant had a CADD v.1.3 (“combined annotation dependent depletion”) score of 31 and was thus classified as being intolerable (**Figure 1**, left) (19). Segregation analysis confirmed the heterozygous carrier status of both parents (**Figure 1**, right).

At this time, immunophenotyping of peripheral blood cells revealed a selective CD19+ B-cell lymphopenia with a nadir of 0.02 x10^9^/L. Immunoglobulin G and M levels (IgA deficiency) as well as the results of the stimulated T cell proliferation tests were normal, although the vaccine-dependent reactivity was moderately reduced. Since an ongoing telomere length shortening of hematopoietic stem cells had been previously reported to contribute to the development of cytopenia in *NHEJ1*-deficient cases, we had this parameter examined in the Department of Pediatric Hematology and Oncology, University of Freiburg, Germany (20, 21). Comparison of his telomere repeat copy number with that of reference samples confirmed that the telomeres in his peripheral blood cells were, with a ratio of 0.57, indeed severely shortened, namely to an extent that was below the first percentile (0.61) of a healthy control cohort (n=90).

**Figure 2:**
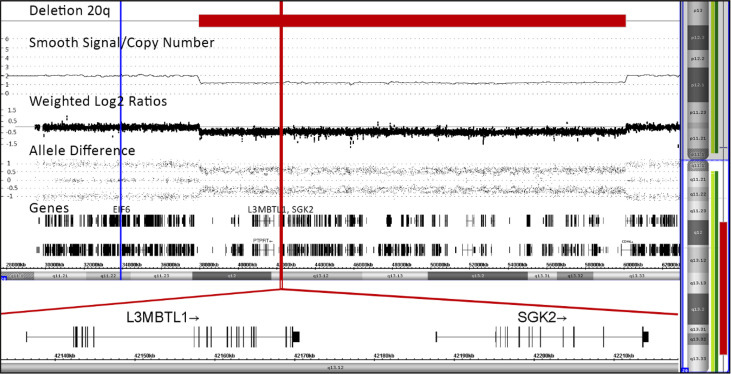
Array pattern of long arm of chromosome 20 showing the interstitial deletion with the following bordering coordinates: chr20:37948298-60071887 (hg19). The deletion extends over 22,124 Mb and encompasses 244 genes, a list of which is provided in the **Supplementary Table 2**. The location of the three potentially most relevant genes, *EIF6, L3MBTL1* and *SGK2*, are indicated with blue and red lines, respectively (24–28). The orientation and detailed structure of the two imprinted ones in the deletion are shown in the blown-up section on the bottom part of the Figure.

To determine the exact location and extension of the hematopoiesis-restricted del(20q), we performed a CytoScan™ HD array analysis. This array comprises 2,670,000 markers, including 750,000 single nucleotide polymorphism probes (Applied Biosystems™, Thermo Fisher Scientific, Waltham, MA, USA). We obtained the data from a commercial service provider and analyzed them in-house with the Chromosome Analysis Suite (ChAS; Applied Biosystems™, ThermoFisher Scientific) software package version 4.1 as described previously in detail (22). Overall, six percent of the autosomal genome was homozygous, indicating consanguinity in the family. In addition to the del(20q) we noted seven such consanguinity-associated homozygous regions that were larger than 3Mb, namely on chromosomes 2(q34-q37.2), which contained the *NHEJ1* pathogenic variants, 3(p12.3-q13.31), 7(q34-q36.1), 8(q22.1-q22.3), 9(p21.3-q21.32), 14(q11.2-q12) and 21(q11.2-q21.3) as well as a unique 78kb duplication at chromosome 20(p11.2) that partially disrupted the *GINS1* gene (**Supplementary Table 2**). Biallelic loss of function pathogenic variants in this gene cause another very rare, phenotypically very similar immunodeficiency syndrome (IMD55; OMIM #617827) with intrauterine growth retardation, chronic neutropenia, and natural killer cell deficiency (23). The 22,124 Mb large interstitial del(20q) encompassed nearly the entire long arm and removed 244 genes that are contained in this region (**Figure 2**). Of the three genes that are of specific relevance in the context of an acquired del(20q), two, namely the imprinted *L3MBTL1* and *SGK2* genes were deleted, whereas the *EIF6* gene was not (**Figure 2**) (24–28).

## Discussion

The case presented herein is only the second NHEJ1-deficient patient who reached adulthood without transplantation (29). Despite the early onset of a refractory cytopenia with a transient monosomy 7 and the emergence and subsequent expansion of a del(20q) clone many years later, his disease neither progressed into a *bona fide* myeloid malignancy nor did it require any specific therapeutic care during the now overall 20 year-long observation period. Given that even his genetic condition and his hematologic disease alone would have sufficed to transplant him, the lack of a suitable donor was in retrospect a stroke of luck for both the patient as well as the treating physicians (11, 14, 30, 31).

The *NHEJ1* gene encodes one of the components of the principle nonhomologous end-joining repair pathway, whose other constituents are the products of *LIG4* (encoding DNA ligase IV), *PRKDC* (encoding DNA-PKcs), *DCLRE1C* (encoding Artemis), and *XRCC4* (encoding XRCC4) (32–35). This system is not only responsible for the repair of double strand breaks but also for the appropriate execution of V(D)J recombination (32–35). Thus, pathogenic variants in any of these genes increase the radiosensitivity of affected tissues and disturb V(D)J as well as class switch recombination processes. The ensuing problems produce developmental defects in form of a growth delay, microcephaly and dysmorphic facial features as well as various types of (severe) combined immunodeficiencies with differing degrees of B and T cell lymphocytopenia (9, 29, 32–37). In addition, such germline pathogenic variants also predispose affected individuals to the development of autoimmune diseases, lymphomas, bone marrow failure as well as lymphoid and occasionally also myeloid leukemias (9, 34, 38, 39). Although we did not perform any functional assays or radiosensitivity studies, the elevated chromosome breakage in the second DEB test at least provides some evidence that the double strand breakage repair was indeed impaired.

Since the overlapping actions of the *PAXX* and *ATM* gene products can to some extent compensate functional impairments of the NHEJ1 protein, one would not expect that it plays such a vital role as, for instance, that of the *LIG4*-encoded DNA ligase IV (34, 35, 40, 41). Nevertheless, NHEJ1 deficiencies still affect the respective repair and recombination processes quite profoundly, so that the ensuing clinical consequences usually resemble those of the otherwise more severe *LIG4* defects (34). Less than 50 cases with bi-allelic *NHEJ1* loss-of-function pathogenic variants have so far been documented in the literature (21, 29, 31, 32, 36, 37, 42–44). The heterogeneous phenotypes and variable clinical courses of patients with different but also identical pathogenic variants severely impede any attempts to establish an even only approximate genotype-phenotype relationship, not least also because the effects of the diverse pathogenic variants are also cell type-specific and differentiation stage-dependent (29, 42, 45). Cases in point are, for instance, the progressive lymphocytopenia as well as bone marrow aplasia in some NHEJ1*-*deficient individuals, which almost certainly can be put down to a premature aging of hematopoietic stem cells (21, 45, 46). This problem is most likely triggered by the inability of the affected stem cells to properly repair continuously accumulating double strand breaks as well as by a pathogenic variant-triggered decrease in telomerase activity that, as also seen in our patient, leads to a gradual loss of telomeres (21, 45, 46). We are aware of altogether five patients with such a bone marrow aplasia, all of whom were transplanted and, all but one, were alive at the time of reporting (21, 31, 32).

Abnormalities of chromosome 7 are the most common acquired genetic changes in childhood myelodysplastic syndromes. They comprise the loss of an entire copy as well as various structural abnormalities in form of deletions, translocations and isochromosomes of its long arm (14, 47). A monosomy 7 is seen in virtually all types of IBMF, but the frequency of its occurrence varies depending on the underlying primary germ line defect (14, 47). The two most common ones are the SAMD9/SAMD9L and GATA2 syndromes (6, 7, 14). Together they account for at least 50% of pediatric MDS with monosomy 7, although the disease emerges primarily in younger children in the former and primarily in adolescent ones in the latter (6, 7, 14). Moreover, monosomy 7 is also the most common alteration in patients with a hypocellular refractory cytopenia, although it is seen in only approximately nine percent of them (11, 12, 30). The only other case that is vaguely comparable to ours is one with a *LIG4* germline pathogenic variant and an MDS-related deletion of 7q (48).

The emergence of a monosomy 7 in patients with a hypocellular refractory cytopenia usually concurs with a high probability of disease progression. Nevertheless, in some of the patients the abnormal clone may disappear again and thereby lead to a spontaneous improvement or even disease remission. In the meantime, such transient forms of monosomy 7 are well documented in the literature (13, 49–54). In case of SAMD9/9L-associated disorders, monosomy 7 always results from the nonrandom loss of the homologue that carries the respective *SAMD9/9L* germline defect (6, 7, 13, 14, 47, 55, 56). These clones may occasionally experience a spontaneous duplication of the remaining homologue that carries the wild-type *SAMD9/9L*, which will then promote the functional normalization of the bone marrow (6, 7, 13, 14). Since the *NHEJ1* gene is located on chromosome 2 rather than on chromosome 7, we neither expected nor detected such a repair process-associated uniparental disomy 7 in the array analyses and conclude that the monosomic clone had no competitive advantage and simply got lost again (6, 7, 14, 51, 52).

The subsequent appearance and gradual increase of another clone with a del(20q), seven and a half years later, concurred with an improvement of his blood counts as well as with the continuous resolution of the erythropoietic dysplasia. A del(20q), either alone or in combination with other chromosome abnormalities, is seen in many different types of myeloid malignancies of all age groups (57, 58). Incidental observations in non-myeloid malignancies and unexplained cytopenia, however, prove that it is not always a *bona fide* indicator of malignancy and that in such instances the progression to MDS is extremely low (57–59). In children, a solitary del(20q) occurs in an age-dependent manner almost exclusively in those who suffer from a SDS (27, 28, 60–63). With a prevalence of 20% it is also the most common acquired abnormality followed by an isochromosome 7(q10), which is seen in 10% (63). Both these changes may occur either alone, simultaneously, sequentially, or even only transiently, but irrespective of the specific constellation, cases affected by either abnormality hardly ever progress into a genuine myeloid malignancy (28, 63, 64). The cytopenia of SDS patients with a del(20q) remains as stable and the dysplastic alterations as mild as the ones that we observed in our NEJH1-deficient case. The positive influence of a del(20q) on disease development has been put down to the facts that affected totipotent stem cells not only maintain their multipotential differentiation capacity but that, in addition, they also gain a selective advantage (60). However, the impact of such abnormal stem cells in individual settings is virtually impossible to predict, because their destiny is primarily governed by their competitive fitness, which in turn is to a large extent also influenced by a variety of individual host factors and, not least, of course by the type of the preexistent germline defect.

The three genes that are currently in the focus of interest in this context are *L3MBTL1* and *SGK2*, which were lost, and *EIF6*, which was retained in our case (24, 26, 65). *L3MBTL1* and *SGK2* are two paternally expressed imprinted genes that encode a transcriptional repressor and a serine/threonine protein kinase, respectively. These genes are in the minimal commonly deleted region of 30 adult cases with a solitary del(20q), but also lost in SDS patients (26, 28, 57, 66). *In vitro* experiments revealed that their regulatory interactions vary in different hematopoietic lineages and successive stages of differentiation (25, 67, 68). Their coordinated silencing maintains megakaryopoiesis and enhances erythropoiesis but does apparently not equip affected stem and early progenitor cells with any selective advantage (25, 69). Attempts to associate the parental origin of the deletion with their anticipated allele-specific expression patterns produced no clear results and therefore also no coherent picture. Explanations for the various confusing discrepancies ranged from problems that may have arisen from inadequate clone sizes, from admixture of normal cells, from loss of imprinting and, most intriguing, also from the concurrence of two distinct clones, in which one lost the maternal and the other one the paternal allele (25, 26, 67, 69–71). The best clinical, albeit indirect evidence that a clonal del(20q) indeed enhances erythropoiesis derives from polycythemia cases (69). Another notable example relates to the observation that the hemoglobin concentrations and red blood cell counts of SDS patients with a del(20)(q) are higher than those without such a deletion (26).

*EIF6*, the retained gene, encodes the eukaryotic translation initiation factor 6, which is an essential ribosome chaperone protein (65, 72). To allow the formation of the mature 80S ribosome, this factor must first be released from the pre-60S ribosome subunit by the SBDS protein. Thus, *EIF6* inactivating point mutations or deletions become only relevant in patients with preexisting *SBDS* mutations, in whom they will help to reestablish a normal SBDS : EIF6 protein ratio, which improves the maturation and translational capacity of the ribosomes and consequently also enhance the competitive fitness of the affected hematopoietic cells (65, 72). Deletions of the *EIF6* locus in other forms of myeloid malignancies might therefore be functionally irrelevant and merely coincidental.

Taken together, the indolent development of a chromosomally abnormal refractory cytopenia in our patient with an already preexistent severe DNA repair defect is quite remarkable but not a unique phenomenon. Such observations reinforce the growing awareness that the emergence of abnormal clones in IBMF syndromes is not deterministic of malignant transformation. Instead, as we show herein, it can also stabilize and improve the disease process in a quite unexpected manner. The big challenge that derives from this insight is now the need to delineate harmless or even favorable abnormalities from undisputable malignant ones. The definition of even only approximate distinguishing criteria will significantly help to advance clinical decision processes, especially whether and for how long one can rely on a “watch and wait” strategy or whether at all and when one should pursue a more aggressive treatment, such as stem cell transplantation (65, 73–75).

## Data Availability Statement

The raw data supporting the conclusions of this article will be made available by the authors, without undue reservation.

## Ethics Statement

Ethical review and approval was not required for the study on human participants in accordance with the local legislation and institutional requirements. Written informed consent to participate in this study was provided by the participants’ legal guardian/next of kin. Written informed consent was obtained from the minor(s)’ legal guardian/next of kin for the publication of any potentially identifiable images or data included in this article.

## Author Contributions

Conceptualization and supervision: LK, KB, OAH; manuscript drafting and writing: FP, OAH; methodology: RJH, PZ, KN; data analysis and interpretation, RJH, PZ, KN, OAH, KB; provision of clinical information and patient care: FP, WN, MND, LK; illustrations: RJH, PZ, KN. All authors have read and agreed to the published version of the manuscript.

## Conflict of Interest

Authors PZ, KN and OAH were employed by company Labdia, Labordiagnostik.

The remaining authors declare that the research was conducted in the absence of any commercial or financial relationships that could be construed as a potential conflict of interest.

## Publisher’s Note

All claims expressed in this article are solely those of the authors and do not necessarily represent those of their affiliated organizations, or those of the publisher, the editors and the reviewers. Any product that may be evaluated in this article, or claim that may be made by its manufacturer, is not guaranteed or endorsed by the publisher.
